# Long-Term Outcomes of Coronary and Carotid Artery Disease Revascularization in the FRIENDS Study

**DOI:** 10.1155/2019/8586927

**Published:** 2019-06-20

**Authors:** Fabrizio Tomai, Anna Piccoli, Fausto Castriota, Luca Weltert, Bernhard Reimers, Gabriele Pesarini, Raoul Borioni, Giovanni De Persio, Roberto Nerla, Andrea Pacchioni, Alberto Cremonesi, Flavio Ribichini

**Affiliations:** ^1^Cardiovascular Department, European Hospital, Rome, Italy; ^2^Division of Cardiology, University of Verona, Italy; ^3^Cardiovascular Department of Humanitas Gavazzeni Hospital, Bergamo, Italy; ^4^Division of Cardiology, Istituto Clinico Humanitas, Rozzano, Italy; ^5^Division of Cardiology, Ospedale Civile di Mirano, Italy

## Abstract

**Objectives:**

The aim of this study is to assess long-term-outcomes of patients with concomitant CAD and COD treated with different revascularization strategies.

**Background:**

Multisite artery disease is common and patients with combined disease have poor prognosis. The best therapeutic strategy for patients with concomitant carotid obstructive disease (COD) and coronary artery disease (CAD) remains controversial.

**Methods:**

This observational registry enrolled, between January 2006 and December 2012, 1022 consecutive patients from high volume institutions with concomitant CAD and COD suitable for endovascular, surgical, or hybrid revascularization in both territories selected by consensus of a multidisciplinary team.

**Results:**

The cumulative incidence of 5-year major cardiovascular events (MACCE) including cardiovascular death, myocardial infarction (MI), or stroke in the overall population was 12%. The incidence of 5-year MACCE was not statistically different in the surgical, endovascular, or hybrid patients group (10.1% vs. 13.0% vs. 13.2%, P = .257, respectively). However, the hybrid group exhibited rates of myocardial infarction, chronic kidney disease, and cumulative incidence of all clinical events higher than the surgical group. After propensity score matching, the incidence of 5-year MACCE was similar in the three groups (13.0% vs. 15.0% vs. 16.0%, p = .947, respectively).

**Conclusions:**

An individualized revascularization approach of patients with combined CAD and COD yields very good results at long-term follow-up, despite the high risk of this multilevel population even when the baseline clinical features are equalized.

## 1. Introduction

Multisite artery disease (MSAD) is defined by the simultaneous presence of clinically relevant atherosclerotic lesions in at least two major vascular territories [1]. Patients with MSAD are regularly encountered in clinical practice and their prognosis is poorer than that of patients with just one territory affected [2–5]; however, recommendations for the treatment of such patients are inconsistent. Indeed, in general the treatment strategy is decided case by case within the context of a dedicated multidisciplinary team and most experts agree on focusing first on the symptomatic vascular territory. In particular, patients with coexisting coronary artery disease (CAD) and carotid obstructive disease (COD) represent a complex and high-risk population, in whom revascularization can be performed by surgical, endovascular, or hybrid strategies (the latest being a combined approach that includes both forms of treatment).

The absence of dedicated randomised trials or large registries designed to assess advantages, shortcomings, and long-term-outcomes of patients with concomitant CAD and COD treated with different revascularization strategies add further uncertainty on the management of this rapidly growing subset of high-risk patients [6].

This is the background to the FRIENDS (Finalized Research in ENDovascular Strategies) working group that devised an observational study, whose aim is to assess clinical outcomes of a wide population with concomitant CAD and COD disease treated according to the “best standard of care”. The FRIENDS observational registry gathered data of patients from four high-volume centers skilled for the treatment of MSAD. We previously reported the 30-day and 1-year outcomes of different revascularization strategies in patients with coexistent CAD and COD [7–9]. Here we report the long-term outcome of these patients and a propensity matching of the different treatment groups.

## 2. Materials and Methods

FRIENDS is an Italian, spontaneously generated, independent and no profit working group whose members are engaged at high volume Italian institutions and are committed to cardiovascular care and work with a shared intention under common coordination.

### 2.1. Patient Population and Data Collection

Between January 2006 and December 2012, 1022 consecutive patients with concomitant CAD and COD suitable for endovascular, surgical or hybrid revascularization in one or both territories have been enrolled in the FRIENDS registry. From January 2006 all consecutive patients who satisfied all inclusion and exclusion criteria were enrolled in our prospective registry. The database was designed to collect uniformly all individual dataset from each participating center. All patients included in this study gave informed consent to undergo the proposed treatment and complete the prespecified follow-up program. The ethical committees of each participating institution approved aims and methods of this study under the coordination of the University of Verona ethical committee (CESC no. 2246). Clinical follow-up was obtained prospectively by either clinical visit or telephone contact. The 30-day and 1-year results of this research have previously been published [7–9]. Here we report the results of long-term clinical follow-up in the overall population.

### 2.2. Inclusion and Exclusion Criteria

#### 2.2.1. Inclusion Criteria

- Written informed consent.
- Diagnosis of concomitant CAD and COD with indication to revascularization. All patients, regardless of the treatment strategy applied, should show a significant concomitant vascular disease in both the territories. CAD and COD definitions were previously reported [1, 10, 11]. Briefly, CAD with indication to treatment was diagnosed by selected coronary angiography if a stenosis >70% was present in at least one of the major coronary branches or >50% in the left main; COD with indication to treatment was diagnosed in presence of a stenosis involving the internal carotid artery ≥70% in neurologically asymptomatic patients and ≥50% in neurologically symptomatic patients. When carotid artery stenting (CAS) was indicated, lesion severity was assessed also by selective angiography. Patients were considered symptomatic if an ipsilateral cerebrovascular event (including transient ischemic attack, amaurosis fugax, ischemic stroke, or retinal infarction) had occurred within the prior 6 months.

#### 2.2.2. Exclusion Criteria

- Nonatherosclerotic nature of the disease.
- Lack of informed consent.
- Reduced survival expectancy due to severe comorbidities (<2 years).
- Impossibility to obtain follow-up information.

### 2.3. Revascularization Strategies and Techniques

Revascularization strategies and techniques have been previously published [7–9] and here briefly summarized. Each center appointed a committee of experts in cardiac and vascular medicine (cardiovascular team) that jointly evaluated each case to assess the best therapeutic indication in these specific patient settings [12]. A cardiovascular team includes cardiac and vascular surgeons and clinical and interventional cardiologists. Opinions of neurologists and radiologists were obtained as needed. Coronary and carotid interventions were mandated either by symptoms as well as by the indication consistent with current guidelines [11, 13–15]. Interventions were considered simultaneous when performed in the same procedure or within the same day. Staged interventions were intended as those performed within a range of 1 to 45 days from the first procedure. The sequence of intervention was established by the cardiovascular team considering the neurological and/or cardiovascular symptoms and the anatomical characteristics of the atherosclerotic lesions after diagnostic angiography. Coronary artery bypass grafting (CABG) was performed “on pump” in 70% of cases. Carotid endoarterectomy (CEA) was performed according to conventional techniques preferring local anesthesia when possible. According to standard clinical practice, percutaneous coronary interventions (PCI) was performed via a 6- or 7-F sheath in the femoral or radial artery. Stent placement during PCI followed nearly 100% of balloon dilatation and, in patients with multivessel CAD, the most complete degree of revascularization was attempted unless contraindicated by severe comorbidities or particularly unfavorable anatomy. The carotid artery stenting (CAS) procedures were performed via a 7-, 8-, or 9-F sheath in the femoral artery using different kinds of stents and distal protection devices according to individual clinical and anatomical characteristics as recommended by experts [16].

### 2.4. Antithrombotic Regimen

All patients treated by endovascular procedures received anticoagulation with unfractioned heparin and antiplatelet therapy with aspirin before catheterization. A loading dose of a thienopyridine was administered before percutaneous coronary intervention or CAS, and a dual antiplatelet therapy was advised for a minimum of 1 and maximum 12 months according to the clinical presentation and the type of stent implanted, as recommended by current guidelines [11, 13]. In patients needing chronic oral anticoagulation the antithrombotic regimen was managed according to the CHA2DS2-VASc score [17]. In general, a peri-intervention short bridge with low-molecular weight heparin was associated with antiplatelet therapy maintained by one month only, followed by an association of warfarin and aspirin, or dual antiplatelet therapy thereafter.

For surgical candidates, CEA and CABG were performed under aspirin alone, and in patients needing chronic oral anticoagulation the same bridging strategy described before was implemented without association of a thienopyridine. In the hybrid group, patients undergoing percutaneous coronary intervention after CEA started the dual antiplatelet therapy 48 hours after surgery. Those undergoing CABG after CAS in a simultaneous approach were pretreated with aspirin and unfractioned heparin and received a bolus of the short-term IIb-IIIa glycoprotein inhibitor tirofiban during the procedure and the iv infusion was continued until 6 hours before cardiac surgery. If CABG followed CAS in a staged procedure, cardiac surgery was performed after 1 month of dual antiplatelet therapy, under aspirin alone, and 5 days after thienopyridine suspension.

### 2.5. Study Endpoints

Prespecified primary outcomes measure of the analysis was the 5-year incidence of major cardiac and cerebrovascular events (MACCE), including cardiovascular death, myocardial infarction (MI), or stroke, according to the three different revascularization strategies. Secondary endpoints were the 5-year incidence of the individual components of the primary endpoint, any death and chronic kidney disease (CKD) or need of hemodialysis. Endpoints definitions were previously reported [7–9]. In particular myocardial infarction included all spontaneous MI diagnosed by an increase in biomarker values above the upper limit of normal (creatine kinase-myocardial band fraction or, preferably, cardiac troponin), reinfarction (defined as recurrence of symptoms together with ST-segment elevation or new left bundle branch block and an increase in cardiac enzymes after stable or decreasing values), or periprocedural MI (diagnosed by elevation of cardiac biomarker values in patients with normal baseline values). After August 2012, all the MIs were reclassified, and the new one accounted according to the third universal definition of MI [18]. Chronic kidney disease was diagnosed according to the National Kidney Foundation criteria, based on the calculated glomerular filtration rate [19]. Events included in the endpoints were adjudicated by the same independent expert for all the centres to be assessed in a blinded way.

### 2.6. Statistical Methods

Continuous data are expressed as means and standard deviations. Categorical data are presented as absolute values and percentages. Differences between endovascular versus either the surgical and the hybrid group were assessed with Unpaired Students' t-test or Kruskal-Wallis test for continuous variables and with Chi-square test for categorical variables. Power estimate analysis was preliminary carried out considering hypothesis of equivalence of Hazard Ratio at Cox Proportional Hazard Analysis for primary endpoint, with a standard Type 1 error rate (alfa) set af 5% and Power (1-Beta) set at 80%, noninferiority margin (delta) of 0.5, overall Probability of Event (PE) at 0.8, and Hazard Ratio (Theta) at 1, resulting in a total sample size of 217 patients.

Time related event endpoints were assessed by Kaplann-Meier curves and log rank test. Time zero for all time-to-event analyses was the time of the first procedure performed, either coronary or carotid revascularization.

A Cox regression, univariate first and then multivariate, was performed on primary end point including all baseline parameters stated in Table 2 and kind of treatment to establish the impact of each factor (Table 4).

As the three subgroups differed significantly regarding many clinical variables, a propensity score analysis was additionally used to reduce confounding factors between categories. The scoring itself was produced by means of a logistic regression between treatment and all baseline clinical characteristics stated in Table 1; best neighbour method was then used to obtain a one to one, one hundred patients group per each of the three category of population. Efficacy of the process was then tested again and proved satisfactory as shown below.

The association between clinical and treatment variables and MACCE at 5-year follow-up was assessed by means of a Cox proportional hazards regression analysis.

A probability value of less than or equal to 5% was considered significant. SPSS (version 21, IBM Corporation, Somers, New York) and Excel (Microsoft, Redmond, Washington) were used for data analysis.

## 3. Results

From January 2006 to December 2012, 1022 consecutive patients with combined CAD and COD were enrolled in the four hospitals being part of the FRIENDS study group. All clinical and procedural data were prospectively entered in the FRIENDS database and retrospectively analysed. Clinical diagnosis at admission are summarized in Table 1. Descriptions of type of treatment applied were previously published [9] and the comparison of unadjusted baseline clinical characteristics of the patients included in the three different treatment groups is summarized in Table 2.

Follow-up was completed in 92% of patients. Median follow-up was 62.1 months (interquartile range 20.9 months), with 554 of the patients (54.2%) being followed up for at least 5 years. Patients received OMT including statins at maximum tolerated dose, beta-blockers, and ACE inhibitors or angiotensin receptor inhibitors in the majority of cases (respectively, 81%, 74%, and 65%); only 12% of patients were treated with nitrates to control symptoms. The mean systolic blood pressure at follow-up was 134±15 mmHg with no significant difference between the 3 groups.

The cumulative incidence of 5-year MACCE in the overall population was 12%. Specifically, cardiovascular death, MI, and stroke occurred in 7.4%, 4.7%, and 2.2% of patients, respectively. The overall incidence of events was 21.2%, including any death (13.6%), CKD or hemodialysis (7.0%), MI (4.7%), and stroke (2.2%).

### 3.1. Unadjusted Comparison of All Three Strategies

**Figure 1:**
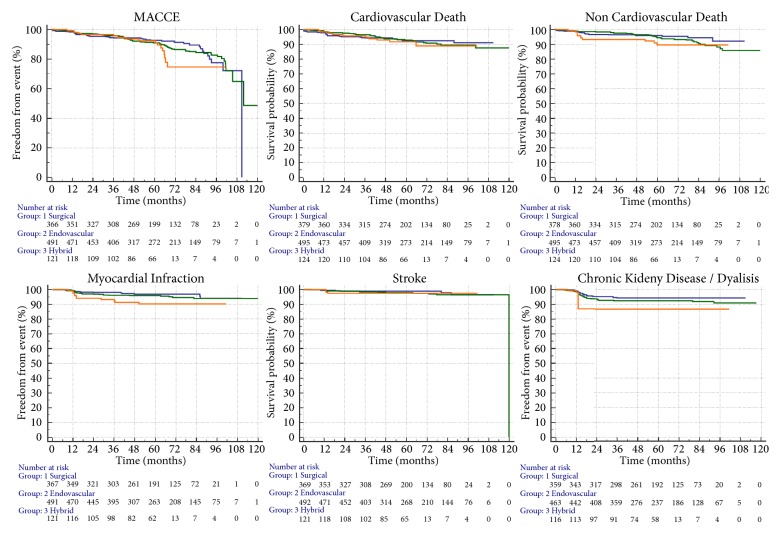
Kaplan-Meier curves for the 5-year primary and secondary endpoints. The Kaplan-Meier curves for the 5-year primary and secondary endpoints are shown for the 3 approaches. Blue lines represent the surgical group (1), green lines the endovascular group, (2) and orange lines the hybrid group (3).

Unadjusted comparison of primary and secondary endpoints for the 3 groups is reported in Table 3. The incidence of 5-year MACCE was not significantly different in the surgical, endovascular or hybrid patients group (10.1% vs. 13.0% vs. 13.2%, P = .257, respectively). However, the hybrid group exhibited rates of myocardial infarction and CKD/haemodialysis higher than the surgical group (hazard ratio 2.98, 95% confidence interval 1.12 to 7.95, and P = .02; and hazard ratio 2.54, 95% confidence interval 1.16 to 5.56, and P = .02; respectively). Also the cumulative incidence of all clinical events was higher in the hybrid group than in the surgical group (hazard ratio 1.9, 95% confidence interval 1.2 to 3.2, and P = .006). Although not statistically significant, the incidence of all clinical events was higher in the hybrid group also when compared with the endovascular group (hazard ratio = 0.7, 95% confidence interval 0.4 to 1.1). No other significant difference was detected among groups. Of note, patients undergoing a hybrid revascularization appeared at higher risk than the other two groups; in fact, they had higher rates of bilateral COD, multivessel CAD, acute coronary syndromes, symptomatic carotid stenosis, and lower left ventricle ejection fraction as shown in Table 2. The Kaplan-Meier curves for the 5-year primary and secondary endpoints are shown in Figure 1.

Univariate and multivariable Cox regression analyses of potential factors affecting the primary end-point have been performed. At univariate model age, left ventricular ejection fraction and the presence of a diffused disease (bilateral COD and multivessel CAD) were significantly associated with the primary endpoint. Multivariable analysis confirmed only age and left ventricular ejection fraction ad independent predictors of the primary end-point.

### 3.2. Propensity-Adjusted Three-Group Comparison for the Primary and Secondary Endpoints

After propensity score matching, three groups of 100 patients each were selected. The adequacy of the propensity score is confirmed in Table 5, showing that patients of the three groups had similar baseline characteristics. The incidence of clinical events at 5 years and the comparisons among groups in the propensity-matched population are shown in Table 6.

**Figure 2:**
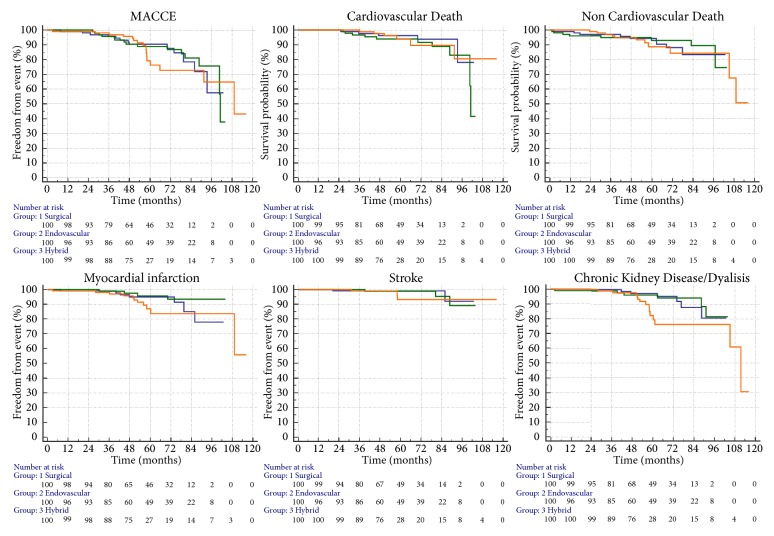
Kaplan-Meier curves for the 5-year primary and secondary endpoints after propensity score matching. The Kaplan-Meier curves for the 5-year primary and secondary endpoints after propensity score matching are shown for the 3 approaches. Blue lines represent the surgical group (1), green lines the endovascular group, (2) and orange lines the hybrid group (3).

The incidence of 5-year MACCE was similar in the surgical, endovascular, or hybrid patients group (13.0% vs. 15.0% vs. 16.0%, p = .947, respectively). Similarly, no significant difference was detected among groups at the single events analysis. However, although not statistically significant, the incidence of myocardial infarction and CKD/hemodialysis was still higher in the hybrid group (11% and 14%, respectively) than in the surgical (7% and 6%, respectively) or endovascular group (4% and 6%, respectively). The Kaplan-Meier curves for the 5-year primary and secondary endpoints after propensity score matching are shown in Figure 2.

## 4. Discussion 

Multisite artery disease is a severe manifestation of atherosclerosis that involves simultaneously different vascular territories and therefore the function of multiple organs with a consequent negative impact on the quality of life and survival of affected patients. During the last decades, diagnosis of concomitant atherosclerotic disease in different vascular beds continued to increase because of the prolonged life expectancy, and nowadays patients affected by MSAD represent a daily challenge. The appropriate treatment approach to this disseminated form of atherosclerosis, often associated with several comorbidities, is neither obvious nor easy. Due to the lack of randomized trials and dedicated guidelines, treatment strategies are not standardised and largely depend on expert's consensus, local standards, and habits. Immediate and long-term results even in the best circumstances are strongly dependent on the baseline clinical characteristics that are largely variable in such a complex population.

The FRIENDS study provides a unique opportunity to assess the long-term clinical outcome of a large cohort of patients with MSAD treated according to the best available standards of care and in agreement with the most recent and robust recommendations. The principal observations derived from this study can be summarized as follows.

The long-term incidence of MACCE, ranging between 10.1% and 13.2% at 5 years, is remarkably low despite the advanced age and the high-risk baseline clinical features of this series. These results are particularly evident if comparing them with the long-term prognosis of patients with only one territory affected. A review from Giannopoulos et al. [20] reported a 5-year all-cause mortality of 22.7% in patients with symptomatic COD (>50%) after CEA while in the FRIENDS population did not reach 14%. Brott et al. recently published long-term results of the CREST trial [21] showing an incidence of MACCE of 10% in the surgical group and 12% in the endovascular group after treatment of isolated COD at a median follow-up of 7.4 years. However, it should be remembered that the capture of adverse events may be very different between registries and clinical trials; therefore these results are not fully comparable.

Compared to the expected natural clinical evolution of patients included in our study, the low global event rates suggest an effective protective effect of the revascularization techniques and the medical therapy applied thereafter.

Further analysis of the global results indicates that independently of the revascularization strategy, all patients derived similar benefits despite the evident differences in the baseline risk profile. Noteworthy, only age at enrollment and left ventricular ejection fraction, but not the kind of treatment, were independently associated with the primary end-point at multivariate analysis. Indeed, although patients selected for a hybrid strategy were at a higher clinical risk, the long-term outcome is equalized compared to the immediate and one-year results previously reported [7–9], confirming the long-term benefits of surgery, in particular CABG in reducing MI and mortality in patients with diffuse CAD. Although some unfavorable outcomes in the highest-risk subgroups persist at 5 years, in particular in terms of MI and worsening of the renal function, these do not attain statistical significance supporting the appropriateness of the initial therapeutic choice.

A prespecified accurate propensity analysis was performed in order to better understand the prognostic impact of the revascularization strategies, regardless of the clinical presentation. As expected, when the baseline clinical features were equalized, the clinical outcomes of the three treatment strategies appear fairly equivalent. In support of this interpretation, most MACCEs occur after three years of treatment, as they were not directly related to the treatment technique but rather to aging and its unavoidable consequences (Figures 1 and 2).

The correct management of patients with multi-level vascular disease is particularly challenging and should be tailored according to a comprehensive medical evaluation and local experience rather than focusing on a determined form of intervention [22]. In the vast majority of cases the “leading organ” is the heart, whereas asymptomatic carotid stenoses are often an incidental finding during routine duplex ultrasonography as part of the global patient evaluation [23, 24]. Similarly, even after effective treatment of the COD, the survival of patients with vascular disease is dictated by cardiac events and renal function, and therefore the treatment of CAD may be more determinant in the long-term survival of these patients. Patients with bilateral severe COD undergoing CABG have an augmented risk of perioperative stroke irrespective of the neurological symptoms, and preventive carotid revascularization is encouraged [22, 25]. Our data confirmed that there are no differences between surgical and endovascular carotid treatment in terms of risk of stroke even in a high risk and old population as this. A thoughtful treatment strategy suited to each single case is mandatory in these complex clinical settings, and in addition to our previous reports focused on the immediate and 1-year clinical outcome, the present data provide information on the long-term outcome. These are important results that should be weighed in the decision-making process.

However, this is a multicenter experience and the individual treatment strategy was chosen according to the institutional expertise that derives from consolidated methods applied in high-volume centers committed to the treatment of MSAD with a dedicated multidisciplinary team. Such circumstances can impact clinical outcomes and may not be comparable to experiences obtained elsewhere. As already said, the study results are observational and influenced by the different clinical presentation of each treatment group.

In conclusion, surgical, endovascular, and hybrid treatment of CAD and associated COD yield good and similar long-term clinical results at 5 years follow-up provided that the best-suited revascularization strategy is discussed in a multidisciplinary context, chosen according to the clinical characteristics of each single case and performed with expertise. The lowest all-cause mortality rate was observed in the surgical group; a very low stroke rate was observed in all groups; and a higher myocardial infarction risk was observed among patients who received a hybrid treatment. Five-year outcome, however, was similar among the three groups and compare favorably to the natural history of the disease analyzed in large observational registries [5, 21, 26].

## Figures and Tables

**Table 1:** Clinical diagnosis at admission.

STUDY POPULATION	n= 1022
CARDIOVASCULAR DISEASE	862 (84.3%)

*Acute*	227 (22.2%)

Non-ST segment elevation myocardial infarction	124 (12.1%)

Unstable angina	88 (8.6%)

ST-Elevation Myocardial Infarction	15 (1.5%)

*Chronic*	635 (62.1%)

Stable angina	561 (54.8%)

Indication to CABG	6 (0.6%)

Others	68 (6.7%)

NEUROLOGICAL DISEASE	181 (17.7%)

*Acute*	23 (2.3%)

Transient ischemic attack	10 (1.0%)

Stroke	13 (1.3%)

*Chronic*	158 (15.4%)

Symptomatic*∗*	84 (8.2%)

Asymptomatic (elective Percutaneous Transluminal Angioplasty)	74 (7.2%)

Values are n (%). *∗*neurological symptoms/signs that had not reached criteria for TIA/stroke but led the patient to neurological evaluation. CABG= Coronary Artery Bypass Graft.

**Table 2:** Comparison of baseline clinical characteristics of the three groups.

	Surgical	Endovascular	Hybrid	*p*
	n=391	n=502	n=129	
Age (yrs)	70.8±8.6	73.1±8.0	71.5±8.1	.001*∗*

Male gender	296 (75.7%)	367 (73.1%)	90 (69.8%)	.62

Hypertension	341 (87.2%)	442 (88.0%)	107 (82.9%)	.24

Diabetes	168 (43.0%)	168 (33.5%)	46 (35.7%)	.01*∗∗*

CKD	118 (30.3%)	112 (22.4%)	24 (18.4%)	.01*∗∗*

Left ventricle ejection fraction	56.1±8.0	54.1±8.6	51.3±8.9	.001¶

Bilateral COD	30 (7.7%)	97 (19.4%)	42 (32.9%)	.001*∗∗*

Multivessel CAD	264 (67.5%)	235 (46.8%)	89 (69.0%)	.001*∗*

Acute coronary syndrome	77 (19.7%)	110 (21.9%)	36 (27.9%)	.23

Neurological symptoms	63 (16.1%)	86 (17.2%)	43 (33.3%)	.001¶

Values are n (%) or mean ± SD. *∗*=Endovascular versus Surgical and Hybrid; *∗∗*=Surgical versus Endovascular and Hybrid; ¶=Hybrid versus Surgical and Endovascular. CAD=coronary artery disease; CKD=chronic kidney disease; COD= carotid obstructive disease.

**Table 3:** Unadjusted comparison of primary and secondary endpoints for the 3 groups.

	Surgical	Endovascular	Hybrid	Surgical vs. Endovascular	Surgical vs. Hybrid	Endovascular vs. Hybrid	P value
	Event rates, n (%)			HR (95% CI)			
Any death	43 (11.3%)	72 (14.5%)	21 (16.9%)	0.8 (0.6-1.2)	0.6 (0.3-1.1)	0.7 (0.4-1.3)	.173

Non CV death	17 (4.5%)	34 (6.9%)	11 (8.9%)	0.7 (0.4-1.2)	0.4 (0.2-1.1)	0.6 (0.3-1.4)	.102

CV death	26 (6.8%)	38 (7.7%)	10 (8.2%)	0.9 (0.6-1.5)	0.8 (0.4-1.7)	0.9 (0.4-1.8)	.829

MI	12 (3.3%)	23 (4.7%)	11 (9.1%)	0.7 (0.4-1.4)	0.3 (0.1-0.9)	0.5 (0.2-1.2)	.019*∗*

Stroke	6 (1.6%)	13 (2.6%)	3 (2.5%)	0.7 (0.3-1.7)	0.6 (0.1-2.5)	0.8 (0.3-3.4)	.669

CKD/haemodialysis	19 (5.2%)	35 (7.1%)	15 (12.5%)	0.7 (0.4-1.7)	0.7 (0.4-1.7)	0.6 (0.3-1.2)	.020*∗*

MACCE	37 (10.1%)	64 (13.0%)	16 (13.2%)	0.9 (0.6-1.4)	0.6 (0.3-1.2)	0.7 (0.4-1.3)	.257

All events	58 (15.9%)	117 (23.8%)	32 (26.5%)	0.8 (0.6-1.1)	0.5 (0.3-0.8)	0.7 (0.4-1.1)	.006*∗*

*∗* = surgical versus hybrid. CKD=chronic kidney disease; CV=Cardiovascular; MACCE = Major Adverse Cardiac and Cerebrovascular Events; MI=Myocardial Infarction.

**Table 4:** Univariate and multivariable Cox regression analysis for the primary end-point.

Variable Name	Cox Univariate HR	Univariate Significance	Cox Multivariate HR	Multivariate Significance
Gender	1.017 (0.568-1.821)	0.954		

Age	1.034 (1.009-1.060)	0.007	1.028 (1.001-1.055)	0.041

Hypertension	0.644 (0.404-1.025)	0.064		

Diabetes Mellitus	1.274 (0.885-1.834)	0.193		

Chronic Kidney Disease	1.353 (0.869-2.106)	0.181		

Left Ventricular Ejection Fraction	0.963 (0.945-0.982)	0.000	0.963 (0.945-0.982)	0.000

Bilateral Carotid Obstructive Disease	1.714 (1.126-2.611)	0.012		

Multivessel Coronary Artery Disease	1.459 (1.003 -2.123)	0.048		

Kind of Treatment (Surgery, Endovascual, Hybrid)	1-206 (0.901-1621)	0.206		

**Table 5:** Baseline characteristics and their distribution among the three groups in the propensity-matched population.

	Surgical	Endovascular	Hybrid	P value
	(n=100)	(n=100)	(n=100)	
Age (years)	72.3±7.6	71.04±6.8	72.41±7.5	.34

Male gender	74 (74%)	70 (70%)	69 (69%)	.71

Hypertension	87 (87%)	85 (85%)	83 (83%)	.73

Diabetes	36 (36%)	32 (32%)	33 (33%)	.82

CKD	16 (16%)	23 (23%)	21 (21%)	.44

Left ventricle ejection fraction	53.5 (7.6)	52.3 (9.8)	52.7 (8.9)	.51

Bilateral COD	25 (25%)	19 (19%)	21 (21%)	.57

Multivessel CAD	67 (67%)	64 (64%)	65 (65%)	.90

Acute coronary syndrome	24 (24%)	22 (22%)	26 (26%)	.80

Neurological symptoms	10 (10%)	9 (9%)	9 (9%)	.96

Values are n (%) or mean ± SD. CAD=coronary artery disease; COD= carotid obstructive disease.

**Table 6:** Propensity-adjusted three-group comparison for the primary and secondary endpoints.

	Surgical	Endovascular	Hybrid	Surgical vs. Endovascular	Surgical vs. Hybrid	Endovascular vs. Hybrid	P value
	Event rates, n (%)			HR (95% CI)			
Any death	14 (14%)	18 (18%)	17 (17%)	0.9 (0.4-1.8)	0.9 (0.5-1.8)	1.1 (0.6-2.2)	.920

Non CV death	9 (9%)	8 (8%)	11 (11%)	1.2 (0.5-3.1)	0.9 (0.4-2.4)	0.8 (0.3-2.0)	.872

CV death	5 (5%)	10 (10%)	6 (6%)	0.6 (0.2-1.7)	0.9 (0.3-2.8)	1.6 (0.6-4.5)	.499

MI	7 (7%)	4 (4%)	11 (11%)	1.9 (0.7-5.3)	0.7 (0.2-2.0)	0.4 (0.1-1.1)	.193

Stroke	2 (2%)	3 (3%)	3 (3%)	0.8 (0.1-4.1)	0.6 (0.1-3.7)	0.8 (0.2-4.4)	.885

CKD/haemodialysis	6 (6%)	6 (6%)	14 (14%)	1.0 (0.4-2.7)	0.5 (0.2-1.2)	0.5 (0.2-1.2)	.093

MACCE	13 (13%)	15 (15%)	16 (16%)	1.0 (0.5-2.1)	0.9 (0.4-1.9)	0.9 (0.4-1.8)	.946

All events	24 (24%)	26 (26%)	30 (30%)	0.9 (0.5-1.6)	0.9 (0.5-1.5)	0.8 (0.5-1.4)	.690

CKD=chronic kidney disease; CV=cardiovascular; MACCE=Major Adverse Cardiac and Cerebrovascular Events; MI= myocardial infarction.

## Data Availability

The data used to support the findings of this study are restricted by the University of Verona ethical committee in order to protect patient privacy. Data are available from Prof. Flavio Ribichini for researchers who meet the criteria for access to confidential data.

## Abbreviations List

CABG: Coronary Artery Bypass Graft
CAD: Coronary artery disease
CAS: Carotid artery stenting
CEA: Carotid endarterectomy
CKD: Chronic Kidney Disease
COD: Carotid obstructive disease
CV: Cardiovascular
MACCE: Major cardiovascular events
MI: Myocardial Infarction
MSAD: Multisite Artery Disease.

## Additional Points

*Limitations*. This is a multicentre experience conducted in high-volume centres dedicated to the treatment of multilevel vessel disease with a dedicated multidisciplinary team. Such circumstances have the potential to impact clinical outcomes and may not be comparable to different experiences obtained in other institutions. Furthermore, the study is an observational report without randomization.
